# Advancement and Innovations in Drying of Biopharmaceuticals, Nutraceuticals, and Functional Foods

**DOI:** 10.1007/s12393-024-09381-7

**Published:** 2024-08-07

**Authors:** Rani Puthukulangara Ramachandran, Mohammad Nadimi, Stefan Cenkowski, Jitendra Paliwal

**Affiliations:** 1 Saint-Hyacinthe Research and Development Centre, Agriculture and Agri-Food Canada, 3600, Boulevard Casavant Ouest Saint-Hyacinthe, Québec J2S 8E3 Canada; 2 Department of Biosystems Engineering, University of Manitoba, E2-376, EITC, 75A Chancellor’s Circle, Winnipeg, MB, R3T 2N2 Canada

**Keywords:** Functional foods, Biopharmaceuticals, Bioactive components, Drying methods

## Abstract

Drying is a crucial unit operation within the functional foods and biopharmaceutical industries, acting as a fundamental preservation technique and a mechanism to maintain these products' bioactive components and nutritional values. The heat-sensitive bioactive components, which carry critical quality attributes, necessitate a meticulous selection of drying methods and conditions backed by robust research. In this review, we investigate challenges associated with drying these heat-sensitive materials and examine the impact of various drying methods. Our thorough research extensively covers ten notable drying methods: heat pump drying, freeze-drying, spray drying, vacuum drying, fluidized bed drying, superheated steam drying, infrared drying, microwave drying, osmotic drying, vacuum drying, and supercritical fluid drying. Each method is tailored to address the requirements of specific functional foods and biopharmaceuticals and provides a comprehensive account of each technique's inherent advantages and potential limitations. Further, the review ventures into the exploration of combined hybrid drying techniques and smart drying technologies with industry 4.0 tools such as automation, AI, machine learning, IoT, and cyber-physical systems. These innovative methods are designed to enhance product performance and elevate the quality of the final product in the drying of functional foods and biopharmaceuticals. Through a thorough survey of the drying landscape, this review illuminates the intricacies of these operations and underscores their pivotal role in functional foods and biopharmaceutical production.

## Introduction

Driven by evolving consumer preferences for convenience, health, and satisfaction, the food product market constantly pushes the envelope of innovation. One area of noteworthy progress has been in functional foods and biopharmaceuticals. These advancements cater to consumers’ inclination toward health-promoting foods that offer potential protection against diseases [1]. Functional foods are those that provide health benefits beyond their basic nutritional value. They can be naturally occurring, nutrient- or ingredient-enriched, and recognized for their diverse health-promoting properties. On the other hand, a nutraceutical is identified as a product extracted or purified from food sources and typically marketed in medicinal forms, which are not commonly associated with traditional food items. The term "nutraceutical" is often used synonymously with functional food, highlighting their shared health-benefiting characteristics. Despite their growing popularity, an acceptable, all-encompassing definition for these terms remains elusive, contributing to their continued interchangeability. The varieties and nuances of functional foods are numerous, ranging from foods naturally containing bioactive compounds to those synthesized to have an increased level of such compounds. The diversity of functional food categories and their examples is further elucidated in Table 1.

**Table 1:** Different categories of functional foods

**Category**	**Definition**	**Examples**
Basic foods	Food/ food products that naturally contain the bioactive	• Carrots with beta-carotene • Oat bran and barley cereals with beta-glucan • Green vegetables rich in lutein • Fruits, tea, and citrus containing neutralize free radical
Processed foods with added bioactive	The bioactive does not exist naturally in the food and is added during processing	• Orange juice with added calcium • Milk with added omega-3 fatty acids • Salmon and other fish oils rich in omega-3 fatty acids • Cheese, meat products (a good source of Conjugated Linoleic Acid (CLA)) • Soy-based foods with Isoflavones: Daidzein Genistein • Yogurt and other dairy products (essential source of Lactobacillus)
Food ingredients synthesized to have more bioactive compounds	The level of the bioactive compounds is modified or concentrated beyond its natural level by traditional breeding, special livestock feeding, or genetic engineering	• Yogurt with increased levels of probiotics • Tomatoes with increased levels of lycopene • Eggs with increased levels of omega-3 fatty acids • Foods fortified with indigestible carbohydrates

Source: [1–6]

Simultaneously, the field of biopharmaceuticals has seen tremendous strides. Biopharmaceuticals, comprising biomolecules such as proteins, nucleic acids, antibodies, enzymes, hormones, and vaccines, have been recognized and utilized for their immense therapeutic potential for decades [7, 8]. However, the efficient preservation and processing of these beneficial products pose a remarkable challenge due to their heat-sensitive nature. Both functional foods and biopharmaceuticals contain bioactive components that, while contributing to their health benefits, are highly susceptible to heat, and therefore, careful selection of drying methods and conditions is required [9, 10].

Drying, an integral part of the food and biopharmaceutical industry, serves as an effective preservation technique, ensuring a longer and safer shelf-life of the products. The drying process, however, must be conducted judiciously to minimize damage to the heat-sensitive bioactive components [9, 10]. The current review aims to amalgamate the research and advancements in drying methods, shedding light on their suitability for different biomaterials, their associated problems, and strategies to preserve the active ingredients in biopharmaceuticals, nutraceuticals, and functional foods.

**Fig.1:**
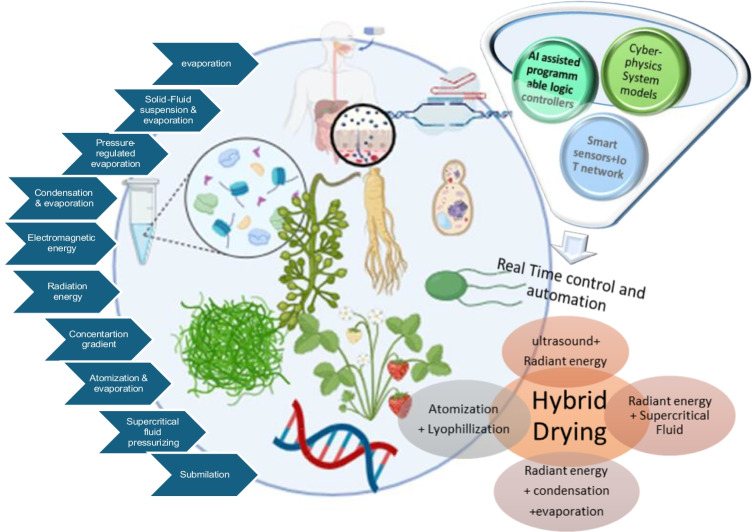
Overview of the review methodology

This review explores the nature of functional foods and biopharmaceuticals, addressing the challenges of drying these heat-sensitive materials. It will examine various drying techniques used to preserve diverse, valuable functional foods and biopharmaceuticals, elaborating on the advantages and disadvantages of each method. The discussion extends to hybrid drying technologies that enhance product performance and quality (Fig. 1). By providing a comprehensive overview of these drying techniques and their applications this review is poised to stimulate further research into developing even more effective preservation methods for these invaluable resources by providing a comprehensive overview of these drying techniques and their applications.

## History of Drying of Sensitive Bioproducts

The world has witnessed the emergence of new technologies in modern medicine and health care, followed by the Second World War. Freeze-dried plasma and antibiotics were the two remarkable medical advances made during wartime. After the discovery of penicillin by Alexander Fleming in 1928, a series of curious investigations were conducted to stabilize pure penicillin. Later, in 1939, Dr. Howard Florey and Ernst B. Chain, working at Oxford, used freeze-drying to stabilize penicillin, earning them the Nobel Prize in 1945 [11, 12]. Later, numerous research studies were done in the field of drying, and many were primarily focused on drying heat-sensitive products. At the end of the 19th century, spray-drying technology emerged, and a patent [13] was issued for spray-drying liquid eggs. The technique proved suitable for drying heat-sensitive biopharmaceutical products as well. With the advent of technology and research, different modifications and designs were studied in various drying methods to improve the quality and safety of dried products, retaining their functional and nutritional properties.

## Major Concerns in Drying of Heat-Sensitive Materials

**Fig. 2:**
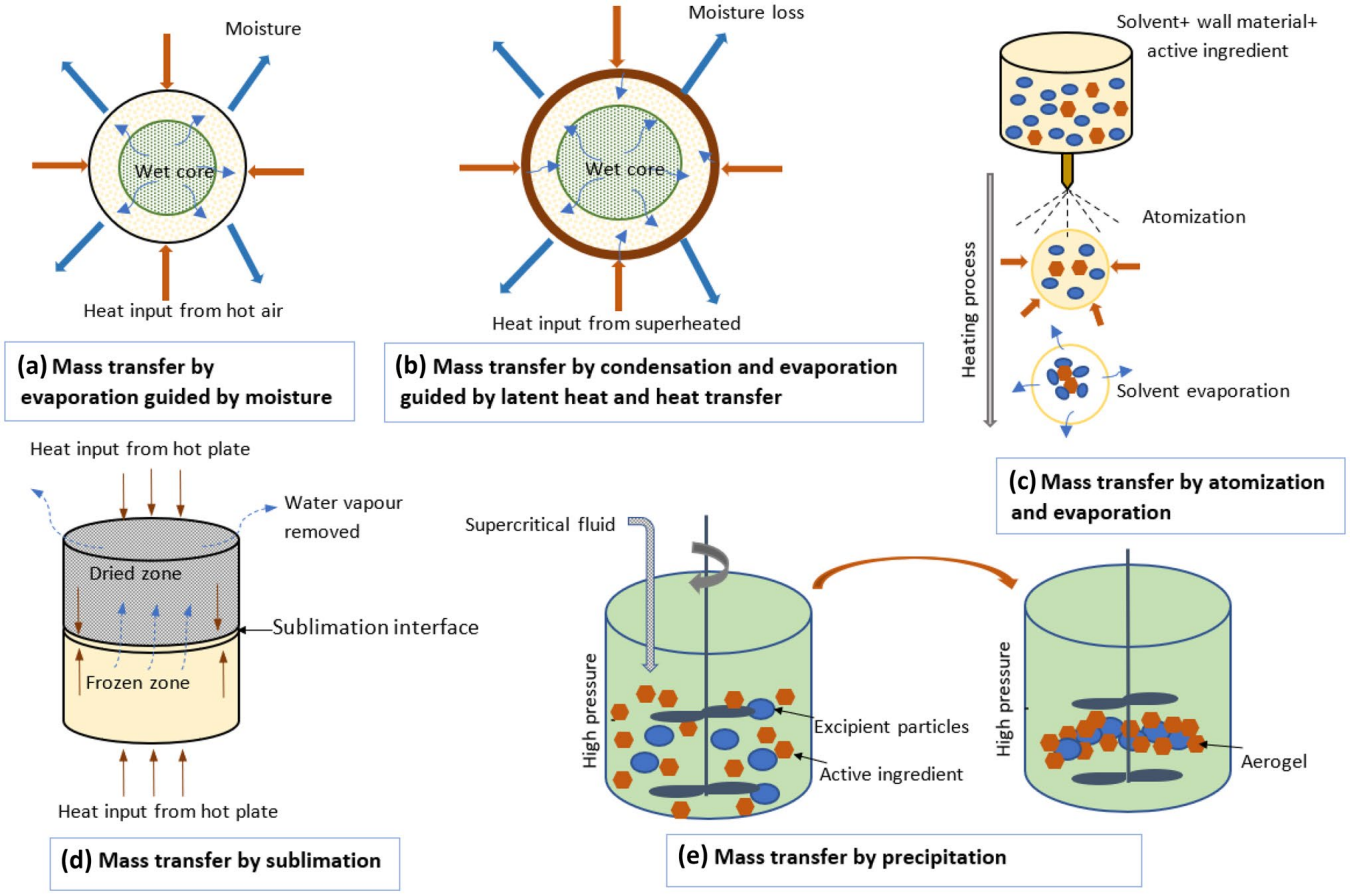
Different mechanisms of removing moisture in the drying process

In the large-scale production of bioactive ingredients for nutraceuticals and functional foods, drying is a critical operation demanding significant energy. As demonstrated in Fig. 2, the removal of moisture in the drying process can occur in different ways: simple evaporation as in hot air or vacuum drying, condensation and evaporation as in superheated stem drying, atomization and evaporation as in spray drying, sublimation as in freeze drying, and precipitation as in supercritical fluid (SFC) drying [14, 15]. Several factors, such as temperature, humidity conditions, pressure, and exposure time, can influence the end product's quality and functionality. Although drying's primary objectives include microbial stability, reducing chemical degradation, facilitating storage, and minimizing transportation costs, researchers also aim to develop drying strategies to mitigate the loss and deformation of bioactive compounds in the dried product [16].

Key indicators of product quality include cellular shrinkage, reduced rehydration capacity, absorbency, solid mobility, surface hardening, and the diminution of volatile aromatic compounds. The evolution of drying methods has led to the categorization of drying technologies into four generations. The first-generation drying technologies, being the most rudimentary, primarily relied on natural elements like sun and wind for drying. With the second generation, artificial heat sources such as ovens and stoves were introduced, greatly improving the reliability and control over the drying process. The third generation brought about mechanized drying techniques, employing hot air ovens, spray drying, and drum drying. This generation saw widespread use in industrial settings, demonstrating enhanced efficiency and control over drying conditions. Currently, we are in the era of fourth-generation drying systems, which use advanced technologies like microwave, infrared, radiofrequency, refractance window, heat pump fluid bed drying, and various hybrid systems. The central aim of these fourth-generation systems is to prioritize preserving food quality, ensuring the retention of essential nutritional components and taste attributes [17–20]. With each progressive generation, the field of drying technology becomes increasingly refined, balancing efficiency, energy consumption, and quality retention.

Drying processes are characterized by conductive and/or convective heat-transfer mechanisms. The primary aim of these processes is to diminish the concentration of residual volatile components in process streams rich in nonvolatile compounds. These procedures facilitate the transfer of energy from the outer surface to the interior of the wet material, resulting in the generation of internal heat within the solid substance. The different types of functional foods, including dairy, meat, grain, and plant-based functional food ingredients, are rich in bioactive elements such as vitamins, essential fatty acids, minerals, antioxidants, etc. These components, however, are highly susceptible to damage from high temperatures. The process of dehydrating these biological molecules may result in substantial chemical, physical, and nutritional degradation, including but not limited to browning reactions, lipid oxidation, colour and aroma depletion, and loss of vitamins and minerals [21].

Certain products are solvent-wet forms that are centrifuged before drying to minimize degradation. However, intense evaporation during drying can still cause a carry-over of solid product particles by the vapour flow. This carry-over can cause clogging of the filters and ducts, resulting in damage to the dryer system. Another common issue with biopharmaceutical and functional products is the agglomeration of particles and their hygroscopic nature, often leading to undesired “lumps” in the end product. Moreover, in the case of organic solids, in which the drying process is controlled by the diffusion of the liquid through the solid, larger lumps lead to longer processing times [8].

Moving forward, the discussion shifts to hybrid drying systems. These represent an advanced frontier in processing heat-sensitive materials. The forthcoming section will delve into these technologies, focusing on preserving the inherent qualities of functional foods and biopharmaceuticals.

## Drying Systems for Heat-Sensitive Biomaterials

As most functional foods, nutraceutical foods, and biopharmaceutical ingredients are thermos-liable with a tendency for structural and functional deformation at extreme drying conditions, selecting the appropriate drying system is key. These drying methods and strategies are also chosen based on the nature of end products, such as food powders, flakes, leathers, or sheets from juices, purees, pastes, or suspensions [10]. Thermal degradation models for various biomolecules and nutrients are essential for understanding and predicting the behavior of heat-sensitive biomaterials under various temperature conditions [22]. Several numerical models aim to describe the kinetics of degradation reactions, assess the impact of temperature on biomaterial stability, and optimize processing parameters to minimize thermal degradation [23, 24]. Thermal degradation of the primary macro-nutrients such as carbohydrates, proteins, and lipids is sometimes essential for converting them to more digestible forms for enhancing nutrient intake. On the other hand, changes to micronutrients such as vitamins, minerals and other functional micronutrients may significantly affect on their functionality and bioavailability [24]. Table 2 summarizes the thermal degradation mechanism and models of macro-nutrients and micro-nutrients. The following section delves into an array of drying methods commonly used in the food and biopharmaceutical industries, specifically focusing on their application in the drying of functional foods and biopharmaceuticals. These methods are critical for preserving the products' quality, nutritional value, and bioactive properties while ensuring safety for consumption or use. The methods discussed include heat pump drying (HPD), freeze-drying, spray drying, vacuum drying, fluidized bed drying (FBD), superheated steam drying, infrared (IR) drying, microwave drying, osmotic drying, and supercritical fluid drying. Each method will be discussed in detail, highlighting its working principle, advantages, limitations, and particular applications in drying functional foods and biopharmaceuticals.

**Table 2:** Thermal degradation mechanism and models of macro-nutrients and micro-nutrients

	Nutrient type	Mechanism and effects of thermal degradation	Models	Reference
Macro-nutrients	Carbohydrate	Mechanism • Temperature < 200 °C -loss of free water and hydroxyl groups in physical and polymeric changes • Temperature 220 °C -550 °C -formation of dehydrated anhydrides with structural and chemical changes • Temperatures > 550 °C further carbonization to degrade to smaller molecules CO_2_, H_2_, CH_4_, etc. Effects o Caramelization of carbohydrates leading to the formation of brown color, aroma and flavor compounds o Pyrolysis of carbohydrates, resulting in the breakdown of complex carbohydrates	The Arrhenius equation and Reaction rate models: *k* = *k*_*0*_ *· e*^*−Ea/RT*^ Ea is the activation energy (kJ mol −1), k is the rate constant, and k_0_ is the frequency/pre-exponential factor. R is the universal gas constant (8.314·10 −3 kJ mol −1 K −1), and T is the absolute temperature (°K) E.g.: Friedman model, Ozawa model, Kissinger model, Flynn-Wall-Ozawa model, Coast-Redfern model	[24–30]
	Proteins	Mechanism • Temperature 100 °C -200 °C -spatial structure changes -thermal aggregation • Temperature > 200 °C – thermal degradation Effects o Denatured protein molecules undergo aggregation or covalent cross-linking, leading to the formation of insoluble protein aggregates o Pyrolysis of proteins, resulting in the breakdown of peptide bonds	First and second-order Kinetic Models Arrhenius model and reaction model	[31–33]
	Lipids	Mechanism • Thermal oxidation 100 ~ 200 °C • Polymerization Effects o Free radicals formation leading to the formation of off-flavors, rancidity, and potentially harmful compounds such as lipid peroxides o Hydrolysis of lipids, resulting in the free fatty acids	Second-order polynomial model Arrhenius model	[24, 34–37]
Micro-nutrients	Vitamins	Mechanism • Thermal oxidation and degradation Effects o Lower bioactivity, irreversible binding to other molecules, or degradation to inactive compounds o 300–500 °C, some vitamins (Vitamin A) decomposes to form aromatic substances	Vitamin C, D, & β-carotene- first-order reaction kinetic $ln\left(\frac{{C}_{t}}{{C}_{0}}\right)=-kt$ Where, *C*_*0*_ -initial concentration of vitamin, *C*_*t*_ – measured concentration of vitamin at time t and *k* temperature- dependent rate constant	[24, 38–42]
	Minerals	Mineral stability and availability are reported to have minimal impact by drying processing than other macro- and micronutrients	-	[43, 44]
	Phenols, Flavonoids and Glycosides	Mechanism • Maillard reaction Effects o Some polyphenols and flavonoids may increase during drying, but long-term exposure of heat reducing their concentration and bioavailability	First-order reaction kinetic	[39, 45, 46]

### Heat Pump Drying

The Heat Pump Dryer System (HPDS) represents an innovative and energy-saving approach to drying and dehydration processes that harnesses low-grade energy to heat the drying medium. Heat pump drying technology is used in high-value foods and biomaterials where low-temperature drying generally ranges from 45 to 70 °C and well-controlled conditions are essential [47]. Its potential as a waste heat recovery system and high drying efficiency have boosted HPD’s popularity [48, 49]. Heat pumps can be classified into different designs, such as gas-engine-driven heat pumps [50–53], ground source heat pumps (GSHP), solar heat pumps [54–56], photovoltaic/thermal (PV/T) heat pumps [57], chemical heat pumps [58], and desiccant heat pumps [59, 60].

This technology is particularly suitable for high-value products, as it allows for controlled transient drying conditions in terms of temperature, humidity, and air velocity, thereby improving product quality attributes and reducing drying costs. HPD has proven to be a reliable method for biomaterials or food materials, including aquatic food products with a high content of phenolics, chlorophyll, ascorbic acid, phycocyanin, and antioxidant activity [48, 61–64]. Some of the advantages of HPD include [53, 65]:

- Lower energy consumption (about 60%) for each unit of water removed, and therefore, higher energy efficiency with improved heat recovery
- Well-controlled temperature profiles, making it highly suitable for heat-sensitive high-value products with better quality outcomes
- Flexibility in drying conditions as it can generate temperatures typically ranging from -20 to 70 °C (with auxiliary heating) and a relative humidity of 15–80% (with a humidification system).

Despite these numerous benefits, HPD also has some limitations. The dryers require regular maintenance of components (compressor, refrigerant filters, etc.), and using CFCs in the refrigerant cycle presents environmental concerns. The technique is also not universally suitable for preserving all bioactive compounds. For instance, HPD can negatively affect ascorbic acid in functional food or biomaterials [62, 66].

Another notable hindrance to the widespread adoption of HPD is the constraints in achievable drying temperatures and the substantial capital required for setting up the system. However, these challenges do not overshadow the key benefits of this technology. Its ability to precisely control the operating temperature and relative humidity makes it ideal for drying functional foods, yielding minimal discoloration and ascorbic acid degradation. Despite the limits on temperature range, this precision positions HPD as a promising technology for enhancing the preservation of high-value foods and biomaterials.

### Freeze Drying

Freeze drying, also known as lyophilization, is primarily utilized to remove water from sensitive biological molecules. This procedure prevents damage, enabling their preservation in a storable state that can be reconstituted simply by adding water. This method is optimal for preserving biopharmaceutical/nutraceutical products (Table 2) like antibiotics, bacteria, vaccines, diagnostic medications, protein-containing, biotechnological products, cells and tissues, and chemicals [67, 68]. Furthermore, freeze-drying is apt for preserving and drying various high-value functional foods like fruits, dairy products, meat proteins, eggs, etc. [69, 70].

Owing to the water being removed in its frozen state rather than its liquid state, the material's morphology, solubility, and chemical integrity are largely maintained after freeze-drying [71]. Freeze-drying is a three-phase process: initially, the product is frozen, decreasing the temperature to cause most of the water to crystallize, leaving only a small fraction unfrozen and incorporated within the product. Subsequently, the primary drying phase occurs, during which the chamber pressure is reduced to enable sublimation while heat is concurrently supplied to the product. The sublimation process is initiated from the material surface, which is driven by the vapor pressure gradient above the sublimation surface *P*_vi_ and the evaporated surface *P*_*a*_ and the rate of sublimation is computed by Eq. 1 [72].

1 $$S=\frac{\left({P}_{iv}-{P}_{a}\right)}{\left({R}_{d}+{R}_{d}+{R}_{i}\right)}$$

where S is the sublimation rate, kg/(m^2^·s); *R*_*d*_ is the resistance inside the dry layer, m^2^/(Pa·s kg); *R*_*s*_ is the resistance to mass transfer from the dry surface to the resublimation surface, m^2^/(Pa·s kg); and *R*_*i*_ is the ice sublimation resistance, m^2^/(Pa·s kg).

And

1a $${R}_{i}= \frac{\sqrt{{T}_{i}}}{{K}_{i}}$$

Under the general assumption that the resistance of the convective mass transfer from the evaporation surface to the resublimation surface is negligible, the maximum sublimation rate possible can be computed as:

2 $${S}_{max}= \frac{{P}_{iv}{K}_{i}}{\sqrt{{T}_{i}}}$$

In primary stage drying, there is a moving interface of freeze-dried layer and frozen layer, and there is no distinctive boundary between the first and second phases of freeze-drying [72]. Equations 3a and 3b give the initial conduction heat transfer from the material surface to the sublimation interface and the frozen layer to sublimation interface, respectively [73].

3a $${Q}_{d}=\frac{2{\lambda }_{d}}{\left(\nicefrac{1}{\sqrt{\pi {A}_{s}}}\right)-\left(\nicefrac{1}{\sqrt{\pi {A}_{ext}}}\right)}\left({T}_{ext}-{T}_{s}\right)$$

3b $${Q}_{i}=\frac{2{\lambda }_{i}}{\left(\nicefrac{1}{\sqrt{\pi {A}_{f}}}\right)-\left(\nicefrac{1}{\sqrt{\pi {A}_{s}}}\right)}\left({T}_{s}-{T}_{f}\right)$$

where ${Q}_{d}$ and ${Q}_{i}$ are the heat flux through dried layer and frozen layer in (W), respectively; ${\lambda }_{d}$ and ${\lambda }_{i}$ are the thermal conductivity of dried layer and frozen layer in (W/mK), respectively; ${A}_{s}$, ${A}_{f}$, and ${A}_{ext}$ are the surface area of the sublimation interface, frozen layer, and external surface in (m^2^), respectively; ${T}_{s}$, ${T}_{ext}$, and ${T}_{f}$ are the temperature of the sublimation interface, external surface, and frozen layer in (K), respectively.

In the secondary drying phase, the product's temperature is increased to remove residual moisture, including bound and unfrozen water [74]. The heat transfer rate by conduction can be defined as the heat flux conducted through the frozen layer of the material (Eq. 3b). This technique has captivated researchers due to its capability to dry materials at lower temperatures, thereby maintaining their original colour, texture, and quality [70, 75]. The application of novel freeze-drying technologies such as Thin film freeze-drying (TFFD) enabled the production of uniform-sized aerosol particles for biopharmaceutical products such as Inhalation-based medication delivery. TFFD has several advantages over traditional freeze-drying processes in biopharmaceutical applications. TFFD uses an intermediate freezing rate, typically between 10^2^ and 10^3^ k/s, which is faster than standard freeze-drying [76]. This intermediate freezing rate improves the structural integrity and bioactivity of sensitive biopharmaceutical compounds enabling the production of engineered dry powders and facilitating precise dosing. Table 3 illustrates the freeze-drying conditions for different foods with bioactive components in FBD.

**Table 3:** Freeze drying conditions for different foods with bioactive components

**Product**	**Drying condition**	**Drying Pressure (Pa)**	**References**
Green banana flours (Starch and crude fibre)	Temperature: ^-^47 to ^-^50 °C	700	[77]
Brazilian ginseng root (beta-ecdysone & fructo-oligosaccharides)	Temperature: ^-^40 °C	Atmospheric	[78]
Symbiotic drink with *lactobacillus casei*	Temperature: ^-^49 °C	1000	[68]
Seabuckthorn berries (phenolic, carotenoids, fatty acids, and vitamin contents)	^-^20 to ^-^50 °C shelf plate temperature	Atmospheric	[79, 80]
Blueberries (polyphenols, antioxidant activity, and ascorbic acid)	Temperature: ^-^30 °C	Atmospheric	[81]
Submicron lactate dehydrogenase (LDH) protein particles	lyophilization (1 K/min) and spray freeze-drying (SFD) (10^6^ K/s), temperature –50 to -140 oC	Atmospheric	[76]
Encapsulated Probiotic bacteria	chamber freeze-drier at -80 oC	0.02 mbar	[82]
Encapsulated Spirulina Maxima in whey protein	Temperature: −50 °C	0.04 mbar	[83]
Monoclonal antibodies formulated with lactose/leucine	Temperature - ^-^100 °C	Atmospheric	[84]

Among the key advantages of freeze-drying for food and biomaterial drying are:

- Preservation of structural, biochemical, and immunological characteristics
- Enhanced viability or activity rates, along with improved textural attributes, owing to drying at low temperatures
- Effective recovery of volatile substances, maintaining structural integrity, surface area, and stoichiometric balances, leading to high product yield, prolonged shelf life, and decreased weight for easier storage, transportation, and handling [85]
- Minimal oxidative reactions due to the absence of oxygen during drying, maintaining the quality of the final product.

However, the broad implementation of freeze-drying is constrained by the significant capital investment required. It is a high-energy, high-cost process for both operation and maintenance. Despite these limitations, freeze-drying remains an effective method for protein powder production. Nevertheless, issues such as ice formation, solute and protein concentration affecting protein stability, and potential cold denaturation during the freezing process are concerns. To address these issues, hybrid techniques such as combined spray- and freeze-drying, thin film freeze-drying, etc., have been developed, which involve spraying the product into a cryogenic medium, followed by the standard primary and secondary drying processes of freeze-drying [86–90].

### Spray Drying

Spray drying, a popular particle formation and drying method, is particularly effective for continuously producing dry solids. These can be either powder or agglomerated particles derived from a liquid feedstock [91, 92]. This technology excels when the final product must meet specific quality standards, such as particle size distribution, residual moisture content, bulk density, and particle morphology.

The spray drying process involves rapid heat and mass transfer as the liquid feed is atomized into fine droplets and introduced into a hot airstream. The water evaporates from the droplet during this process, and the resulting dried powder is cooled and collected using cyclone separators. Spray drying modelling is one of the most commonly simulated models using computational fluid dynamics (CFD) [93]. The advanced computational power of CFD was reported to be effective in solving very sophisticated models such as the continuous phase flow model, droplet agglomeration models, particle droplet tracking, and wall depositions models [94]. The mechanism of increased surface area for evaporation of moisture from the atomized particles is attributed to the uniform and faster drying of spray droplets. Consequently, a single heat transfer equation can be utilized to model the heat flux to the droplet in the heating period and the following wet bulb temperature period (Eq. 4) [95].

4 $$\frac{d{T}_{p}}{dt}=h\left({T}_{g}-{T}_{p}\right)\frac{4\pi {R}_{p}^{2}}{{C}_{pp}{m}_{p}}- \frac{{h}_{l}{m}_{r}}{{C}_{pp}{m}_{p}}$$

where ${T}_{g}$ and ${T}_{p}$ are the drying medium and spray particle temperature in (K), respectively; *R*_*p*_ is the spray particle diameter in m; ${C}_{pp}$ is the specific heat capacity of spray particle in (J/kg⋅K); ${m}_{p}$ represents spray particle mass; ${m}_{r}$ is mass flow rate and ${h}_{l}$ is the latent heat of vaporization in (J/kg).

The spray drying technique has widespread application in the biopharmaceutical industry [96] and in drying of encapsulated food ingredients [97] (Table 3). It is mainly used for microencapsulating the active ingredients of many biological materials, such as flavours, lipids, essential fatty acids, carotenoids, and more. The spray drying technique with microencapsulation was also reported to be a potential solution for manufacturing food additives for food fortification applications such as minerals [98]. The active ingredient is homogenized in an emulsion, which forms the microcapsules' coating. Subsequently, the active ingredient emulsion is spray-dried (Table 4).

**Table 4:** Drying conditions for different encapsulated active ingredients in spray drying

**Encapsulated ingredient**	**Wall material**	**Air inlet temperature (°C)**	**Air outlet temperature (°C)**	**References**
Anhydrous milk fat	Whey proteins/lactose/ Maltodextrin	160	80	[99, 100]
Ethyl butyrate ethyl caprylate	Maltodextrin/gum arabic	160	80	[101, 102]
Caraway essential oil	Maltodextrin/Skim milk powder	175–185	85–95	[103]
Cardamom oleoresin	Gum arabic/modified starch/maltodextrin	176–180	115–125	[104, 105]
Bixin	Maltodextrin/gum arabic/modified starch	180	130	[106, 107]
d-Limonene	Maltodextrin/gum arabic/modified starch	200	100–120	[108, 109]
l-Menthol	Gum arabic	180	95–105	[110]
Black pepper oleoresin	Gum arabic/whey protein concentrate	176–180	105–115	[111, 112]
Cumin oleoresin	Maltodextrin/ gum arabic/modified starch	158–162	115–125	[113]
Arachidonyl l-ascorbate	Maltodextrin/gum arabic/soybean polysaccharides	200	100–110	[114]
Fish Oil	konjac glucomannan, Soybean protein isolate, potato starch	200	80	[115]
Fish oil	Sugar beet pectin/glucose syrup	170	70	[116, 117]
Short-chain fatty acid	Maltodextrin/gum arabic	180	90	[118, 119]
Hawthorn Berry polyphenols	β-cyclodextrin, whey protein isolate, gum arabic	165		[120]
Lycopene	Gelatin/sucrose	190	52	[121, 122]
Turmeric oleoresin	Maltodextrin/gum arabic	150–200	90	[123]

This prevalent method for drying liquid products has numerous advantages, including:

- Drying time is comparatively less than other drying methods since the heat transfer rate is high
- Good reconstitution capacity and product quality
- Minimal chances of thermal denaturation as the droplet's surface temperature is maintained at the wet-bulb temperature, significantly lower than the drying gas temperature
- Enhanced bioavailability of active ingredients and controlled release in encapsulated products
- Improved control over particle size as the feed droplet size can be easily regulated.

However, it's essential to note that spray-dried products are thermoplastic and hygroscopic. As such, product recovery post-drying should be done swiftly and carefully to avoid the product sticking to the dryer walls, which could reduce overall efficiency. Moreover, these dried products are highly sensitive to moisture and temperature fluctuations during storage. Therefore, meticulous efforts must be made to maintain precise relative humidity and temperature levels during storage [124, 125].

### Fluidized Bed Drying

Fluidized bed drying is widely applied in the drying of granular solids in various industries such as food, ceramics, biopharmaceuticals, and for drying phytochemicals like organic acids, carbohydrates, reducing sugars, lipids, and proteins [126–131]. This method is suitable for drying powders in the 50–2000 μm range, thanks to its high heat and mass transfer rates. FBD's effectiveness lies in its fluidization process, allowing for improved drying rates and reduced drying time. The fluidized bed drying process is another multiphase drying model, as the fluid and solid phases are in an interacting continuum. The drying process is governed by the continuum phase heat transfer from the drying medium into the solid phase. Therefore, the general continuum equation for heat, mass and momentum transfer for the fluid medium is set as the boundary conditions for the solid phase drying modelling. The solid phase dying is governed by diffusion equations (Eqs. 5a and 5b) for energy and mass transfer, respectively [132].

5a $$\frac{\partial {T}_{m}}{\partial t}=\frac{{\lambda }_{m}}{{\rho }_{m{C}_{pm}}}\frac{{\partial }^{2}{T}_{m}}{\partial {{R}_{m}}^{2}}$$

5b $$\frac{\partial {M}_{m}}{\partial t}=Deff\frac{{\partial }^{2}{M}_{m}}{\partial {{R}_{m}}^{2}}+\frac{2}{{R}_{m}}\frac{\partial {M}_{m}}{\partial {R}_{m}}$$

where, ${M}_{m}$ is the moisture content of the material in kg/kg; ${T}_{m}$ is the temperature of the material in K; ${C}_{pm}$ is the specific heat capacity of the material; ${\lambda }_{m}$ is the thermal conductivity of the material; ${\rho }_{m}$ is the density of the material kg/m^3^; ${R}_{m}$ is the effective radius of the material in m; *Deff* is the effective diffusivity coefficient m^2^/s.

In FBD, the product is subjected to a high flow velocity greater than its specific gravity. This flow lifts it above the periphery of the dryer mesh. It then decelerates and falls onto an annular zone between the central core and the equipment wall. This flow pattern establishes a unique solid–fluid suspension, ensuring uniform and faster drying [130]. This drying system has several advantages, such as:

- Rapid drying speeds, facilitated by superior contact between gas and particles, results in high rates of heat and mass transfer
- Enhanced thermal efficiency and a reduced flow area in comparison to traditional pneumatic dryers
- Ease of control of the drying process by controlling the fluidization velocity and pressure drop.

However, the method does come with its limitations. It involves high power consumption, requiring suspending the entire bed in the gas phase, leading to a substantial pressure drop. There's an increased chance of attrition and, in some instances, granulation or agglomeration. FBD also has low flexibility for the type of product that can be dried (e.g., it is unsuitable for wet products). Furthermore, frequent issues during the drying of phytochemicals include instability within the drying bed, accumulation of products, coating on non-reactive substances, clumping of particles, and potential system failure. Moreover, there can be losses in bioactive components due to thermal degradation [130]. Therefore, precise control of the drying conditions is necessary for such products with bioactive components, as detailed in Table 5.

**Table 5:** Drying conditions for different foods with bioactive components in fluidized bed drying and superheated steam drying

**Product and bioactive compound**	**Drying condition (air or superheated steam)**	**References**
Phytochemicals	Inlet temperature: 60 to 180 °C Feed flow rate: 3 to 12 g/min	[130]
Green vegetables (broccoli)	Inlet temperature: 60 to 80 °C Particle size: 1-3 cm Air flow rate: 1-3 m/s	[133]
Pellet coated pharmaceuticals	Inlet temperature: 90 °C Gas flow rate: 50 kg/h	[134]
Soybeans	Inlet temperature: 110–140 °C Air velocity: 2.4–4.1 m/s	[135]
Probiotic bacteria	Inlet temperature: 40 °C	[136]
Bee pollen	Inlet temperature: 40 °C Air velocity: 6.0 m/s	[137]
Muskmelon seed	Inlet temperature: 40–60 °C Air velocity: 7–11 m/s	[138]
Wheat grains (dietary fibre and polyphenols)	Steam temperature: 110 to 180 °C	[139, 140]
Fish (omega-3 fatty acid)	Steam temperature: 300 °C Flow rate: 150 kg/h	[141]
Beef (Bioactive antihypertensive peptides)	Steam temperature: 130 to 180 °C Flow rate: 35 -55 kg/h	[142]
Shrimps (carotenoprotein, calcium)	Steam temperature: 120 to 180 °C	[143]
Soybeans (Lysine content)	Steam temperature: < 135 °C Steam velocity: 3.2 m/s	[144]
Oats (beta-glucan)	Steam temperature: 110 to 160 °C Steam velocity: 0.35 to 1.0 m/s	[145]
Waxy rice (Amylose content, Gamma-aminobutyric acid)	Steam temperature: 130–150 °C Steam velocity: 3.5 m/s	[146]

### Superheated Steam Drying

Superheated steam drying is a non-polluting, safe, and energy-efficient method [147–149]. Its capacity to dry materials at temperatures above 100 °C makes it widely applicable in the food industry, as illustrated in Table 5. This method offers various benefits, including reduced drying time and dryer size, ease of integration into production lines, and the ability to recover the energy supplied to the dryer in a usable form.

The drying medium in this method is superheated steam, which operates in a closed cycle, picking up moisture from the wet product in the drying chamber and then condensing the evaporated water in a heat exchanger [150, 151]. Since the drying occurs in a closed environment, the probability of oxidative reaction is minimal, preserving the quality and aroma of the dried material [15, 93]. Moreover, superheated steam drying resembles high-temperature short-time (HTST) treatment in which food gets decontaminated while drying [139, 140]. Low-pressure superheated steam is highly suitable for drying heat-sensitive products like fruits and vegetables, herbs, and other bioactive materials. Low-pressure superheated steam drying takes place in the pressure range of 5–10 kPa [147], at which the steam becomes saturated [152–154]. Compared to hot air-based drying, superheated steam drying has a faster drying rate, as part of the initial accelerated heat transfer is aided by latent heat contributed by the initial condensation and the subsequent free water evaporation (Eqs. 6a and 6b) followed by the diffusion model (Eqs. 5a and 5b) [15, 155].

6a $$\mathrm{If\,T_{m}}<\mathrm{T_{sat}},\frac{d{M}_{m}}{dt}=\frac{{h}_{f}\left({T}_{sat}-{T}_{m}\right)}{{h}_{l}}$$

6b $$\mathrm{If\,T_{m}}\,=\,\mathrm{T_{sat}},\frac{d{M}_{m}}{dt}=\frac{h\left({T}_{ss}-{T}_{sat}\right)}{{h}_{l}}$$

where, *T*_*m*_*, T*_*sat*_*, T*_*ss*_ are the material temperature, saturation temperature and superheated steam temperature in K, respectively; ${h}_{f}$ is the film condensation heat transfer coefficient; *h* is the convective heat transfer coefficient; and ${h}_{l}$ is the latent heat of condensation/evaporation.

Various scholars have utilized superheated steam drying to investigate its ability to preserve bioactive components, primarily antioxidant components, in multiple products. These include tea leaves, where studies have shown significant preservation of antioxidant properties compared to conventional oven drying methods [156], and in other products like onions, where low-pressure superheated steam drying has demonstrated better retention of bioactive components [157]. Another relevant work Suvarnakuta et al. [158] examined the effects of drying methods on the assay and antioxidant activity of xanthones in mangosteen rind. They concluded that hot air drying or low-pressure superheated steam drying at 75 °C is the most suitable drying method to maximize the quantity and quality of mangosteen.

Superheated steam drying has several advantages over hot air drying, including [159–162]:

- Improved drying efficiency when compared to other drying techniques, especially with the closed-loop system.
- Clean process without any emission of flue gases and odor emissions to the environment.
- Absence of direct contact between the product and hot, oxygen-rich gas, reducing the likelihood of oxidation
- Beyond drying, hot steam serves as a sterilizing agent
- Improved control over the drying process by regulating the amount of steam introduced into the compressor, aiding in achieving precise product dryness.

The primary concern with superheated steam drying is the phenomenon known as initial condensation. This occurs when superheated steam comes into contact with a cold solid feed at ambient temperature, leading to vapor condensation on the material surface. This condensed moisture could increase the drying time unless the feed material is preheated by other means. A low-pressure superheated steam system is required to minimize prolonged drying of heat-sensitive bioactive compounds [144]. The full energy efficiency advantage of superheated steam drying can only be fully utilized in a closed-loop system, where the output steam is diverted elsewhere in the processing plant. Such design modifications could add to the system’s complexity and cost [157].

### Infrared Drying

Infrared drying technology uses IR energy directly transferred from a heating element to the food, bypassing the need to heat the surrounding air. Thus, it helps to save energy and drying time. In IR drying, the radiant energy penetrates the product and converts it into heat, heating its surface and inner layers. This intense heating produces a higher heat and mass transfer rate than conventional drying methods. Recent research has highlighted this technique's capability to preserve bioactive components in foods post-drying, showing its effectiveness in maintaining the quality of various food products by preserving their phytochemical content and minimizing the loss of antioxidant activity [163–167].

The drying mechanism is also governed by the diffusion equation as explained in "Fluidized Bed Drying" section, and the energy balance is governed by the conduction, convection and radiation energy as given by Eq. 7 [168]. Lee et al. [169] studied the effects of far-IR drying on the antioxidant and anticoagulant activities of Ecklonia cava (brown seaweed) extracts. Their findings indicated that far-infrared radiation releases and activates low molecular weight bioactive compounds, such as polyphenols, due to its ability to heat materials without degrading their surface molecules [169, 170]. Senevirathne et al. [171] reported that far-infrared radiation drying at 80 °C is an effective and economical method for drying citrus press cakes with minimal loss of antioxidant activity.

7 $${\rho }_{m{C}_{pm}\left(\frac{\partial {T}_{m}}{\partial t}\right)={Q}_{m}-h\left({T}_{m}-{T}_{g}\right)+\zeta \sigma \varepsilon \left({{T}_{r}}^{4}-{{T}_{m}}^{4}\right)-{\dot{m}}_{v}\left({h}_{v}-{h}_{d}\right)}$$

where *T*_*m*_*, T*_*g*_*, T*_*r*_ are the material temperature, drying medium (hot air) temperature, and radiation temperature in K, respectively; ${Q}_{m}$ is the material energy, $\zeta$ is the material shape factor; $\sigma$ is the Stefan-Boltzmann constant, W/cm^2^ ·K^4^; $\varepsilon$ is the emissivity of the material; ${h}_{v}$ is Latent heat of vaporization, J/g; ${h}_{d}$ is the heat of desorption.

IR drying offers several advantages, including:

- Reduced drying time due to higher dehydration rates and high heat transfer rates are achievable with compact heaters
- High energy efficiency
- Rapid process control while maintaining the final product's quality.

The IR drying systems suffer from disadvantages, especially for food and biomaterial drying, such as i) browning reactions resulting in darkening, ii) increased hardness, especially with increased IR power, and iii) deterioration of qualitative parameters [172–174].

Moreover, IR drying is an effective intermittent irradiation method when combined with convective air drying for heat-sensitive materials. An infrared-augmented convective dryer can rapidly remove surface moisture during the initial drying stages, followed by intermittent drying for the remainder of the process. This approach ensures a faster initial drying rate and offers better process control, as the IR power source can be easily cut off in the event of excessively high temperatures in the chamber, preventing overheating of the product. Ratseewo et al. [175] reported that far-infrared radiation drying of pigmented rice enhanced the content of total phenolic, flavonoid, tocopherols, anthocyanins, gallic and ferulic acids, and quercetin compared to traditional hot air drying. Overall, as demonstrated in Table 6, the IR drying method is reported to be appropriate for drying high-valued heat-sensitive food products.

**Table 6:** Drying of different functional foods with bioactive components in Infrared Radiation drying

**Product and bioactive compound**	**Drying condition**	**References**
Ecklonia cava (Brown seaweed) (antioxidants)	Temperature: 40 to 80 °C Optimum temperature: 80 °C	[169, 170]
Citrus press-cakes (antioxidants)	Temperature: 40 to 80 °C Optimum temperature: 80 °C	[171, 176]
Saffron (antioxidants and aroma compounds)	Temperature: 50 to 80 °C Optimum condition: 80 °C for 30 s	[177]
Gamguk flower (herb) (*Chrysanthemum indicum L.*) (phenolic and flavonoid)	Temperature: 50 °C	[178, 179]
Rice hulls (phenolic compounds)	Temperature: 100 °C	[180, 181]
Peanut hulls (antioxidants and radical scavenging activity)	Temperature: 150 °C for 60 min	[182]
Ginkgo biloba seeds (Flavanoids and anti-oxidants)	Temperature: 80 °C	[183]
Garlic (thiosulfinates, phenolic compounds and antioxidants)	Temperature: 50 to 80 °C optimum temperature - < 70 °C	[184]

### Microwave Drying

Microwave drying, or microwave-assisted drying, is a rapid drying technique extensively used in the food industry. This method involves transmitting microwave energy through the product, generating heat due to dipolar polarization and ionic conduction phenomena. This method is distinguished by its volumetric heating, propelled by electromagnetic radiation at 915 or 2,450 MHz frequencies. The heat is generated by the interaction between microwaves and the material, converting a portion of the electromagnetic energy into heat throughout the volume, primarily heating polar molecules like water in the product [185]. The heat transfer mechanism by internal heat generation results in a volumetric heating mechanism of electromagnetic energy supplied by microwaves. The volumetric heat flux is represented by the Eq. 8 [186].

8 $$V\rho {C}_{p}\frac{\partial T}{\partial t}=V\left[\frac{\partial }{\partial x} \left(\frac{\partial T}{\partial t}\right)\right]+P$$

where, *V* is the product volume in *m*^3^**,** and *P* is the Power in *W*, generated by the absorption of Microwave. This microwave power absorbed by water molecules (polar) is converted to heat.

Various studies have explored the potential of microwave drying in producing high-quality end products. The utilization of a two-stage microwave power system, which adjusts the power levels during the drying of functional food products, was proposed by [187, 188]. They proposed that adjusting the power levels of microwave energy (1- 2 kW/kg depending on the initial moisture content) could facilitate higher retention of β-carotene in dried carrots. Microwave-assisted vacuum drying has also been recognized as an appropriate drying method for thermolabile products, including certain foods (e.g., cranberries, carrots, garlic, mushrooms) and biopharmaceutical powders and granules [189]. Condurso et al. [190] found that microwave drying considerably increased the concentration of trisulfides and cyclic sulfur compounds, which contribute to the specific aroma of garlic and possess potent anticancer and chemoprotective properties, in Sicilian garlic compared to hot air drying. Moreover, Berteli et al. [191] studied the microwave vacuum drying process for biopharmaceutical granules and found that it is faster than other drying techniques. They highlighted several benefits:

- Enhanced heat and mass diffusion through biomaterial due to its volumetric heating nature
- Quicker formation of internal moisture gradients, leading to enhanced drying speeds
- Accelerated drying rates achieved without raising the surface temperatures
- Enhanced product quality, making it suitable for heat-sensitive products (such as carrots, garlic, mushrooms).

However, despite these advantages, further research is needed to address specific challenges associated with this method. These include problems such as non-uniform product heating and uneven distribution of the electromagnetic field in a microwave cavity [185] (Table 8).

### Osmotic Drying

Osmotic dehydration is a critical process in drying functional foods such as grapes, berries, tomatoes, carrots, and mushrooms, as it minimizes the loss of functional components [192–196]. The technique operates on the principle of osmotic pressure difference caused by the salt and sugar concentration gradient between the cells of the food product and the surrounding medium. This method minimizes organoleptic and nutritional elements in the product, preserving its flavour and nutritional value [193, 196, 197].

Singh et al. [198] conducted studies on drying carrots by osmotic dehydration using sucrose (50° to 80°Brix) and salt solutions (5 to 15%). They reported that the drying occurs through a simultaneous process of water loss and solute diffusion, effectively drying the food product without excessive loss of nutrients following the Ficks diffusion equation. The osmotic pressure of the drying surface rises until it reach a critical level as the diffusion proceeds, resulting in cell membrane rupture. This facilitates increased cell permeabilization index which is measured by electro-physical measurements [199]. The chemical potential gradient, closely associated with the concentration gradient, represents the force exerted on each penetrant molecule during osmosis and diffusion. Under constant temperature and pressure conditions, the chemical potential (μ) can be described by the following equation [200, 201]:

9 $$\mu ={\left(\frac{\partial {E}_{G}}{\partial n}\right)}_{{T}_{i}, {P}_{i}}$$

where, $\left(\frac{\partial {E}_{G}}{\partial n}\right)$ is the partial derivative of the ratio of Gibbs free energy and number of moles of the penetrant. The chemical potential in a liquid phase as a function of temperature and water activity is determined by Eq. 10 [202].

10 $$\mu =\mu^\circ +RT \,\text{ln}\,{a}_{w}$$

where, $\mu^\circ$ is the standard chemical potential, R is the universal gas constant (J/Kmol), and T and ${a}_{w}$ are the absolute temperature (K) and water activity of the substance in the liquid phase, respectively.

García-Segovia et al. [203] investigated the effect of osmotic dehydration on Aloe Vera, focusing on retaining its immunomodulatory, anti-inflammatory, and antibacterial properties. Their research found that optimal results were achieved when osmotic drying was conducted at lower temperatures, specifically at 40 °C, demonstrating the potential for preserving bioactive compounds during the osmotic dehydration process.

Overall, the osmotic drying process is particularly effective for fruits and vegetables. The technique can dewater these items without compromising their nutritional and functional elements, preserving their inherent health benefits. Moreover, the capability to fine-tune the osmotic solution allows for optimization based on the specific properties of the food product, making it a versatile and efficient drying method. However, the technique has limitations, such as potential changes in texture and the need to remove the osmotic agents from the product after drying, which warrant further research and technological improvements. Also, the diffusion rate of water differs for different materials depending on their composition, geometry, and size, and this limits the drying rates, affecting their nutritional quality and organoleptic properties [199].

### Pressure-Regulating Drying (Vacuum Drying)

Leveraging the universal gas laws, where temperature and pressure are directly proportional, pressure-regulating drying, commonly known as vacuum drying, has gained widespread attention among researchers. By reducing atmospheric pressure, vacuum drying enables water to evaporate at lower temperatures, making it an ideal choice for drying heat-sensitive food products like herbs, curry leaves, and carrots. This approach allows for achieving the desired dryness level without compromising the product's quality, as it operates in a pressure-regulated environment [204–206]. Typically, the operating pressure range varies from a vacuum to close to one atmosphere [151].

Orikasa et al. [207] investigated the effect of vacuum drying on the quality attributes of kiwi fruit. They reported that vacuum drying helps to improve the quality and nutritional value of the dried kiwifruit when compared to hot air drying by retaining l-ascorbic acid, a crucial vitamin. In another relevant study, Šumić et al. [208] noted a remarkable retention of functional elements such as phenols, anthocyanins, and total solids after vacuum-drying frozen sour cherries.

Moreover, the vacuum drying technique offers considerable flexibility and is less costly than freeze drying, making it a preferred choice for many custom or hybrid drying systems to preserve heat-sensitive biomolecules. Examples include vacuum-assisted microwave and vacuum foam drying [209, 210].

However, while vacuum drying offers several advantages, it's important to consider its potential limitations. Some challenges include the need for specialized equipment to maintain a constant vacuum, potential issues with oxidation, and slower drying times due to reduced pressure. Despite these challenges, the potential for high-quality dried products positions vacuum drying as an attractive method in the food industry. Continuous research and technological improvements can help address these challenges, increasing the efficiency and applicability of this method.

### Supercritical Fluid Drying

Supercritical fluid drying is a relatively new drying method utilized in the food and biopharmaceutical field, especially in the drying of proteins [211–215]. This technique leverages the anti-solvent properties of SCFs to induce protein precipitation and remove water from formulations. SCFs, existing at temperatures and pressures beyond their critical points, exhibit distinctive characteristics of both liquid and gas states. Their density can exceed that of a liquid under increased pressure, yet they maintain the diffusivity and viscosity similar to a gas, facilitating effective mass transfer. When subjected to a supercritical jet of cosolvent, it dissolves the free water in the material and as penetrates deeper to dissolve entrapped water and bound water. The convective mass transfer is driven by the concentration gradient of cosolvent between the material surface and the fluid medium. This mechanism has it’s disadvantages as there is a high risk of removing water-soluble nutrients and bioactive compounds along with the water. Hence, solvent pressure, flow rate, temperatures, etc., impact the techno-functional properties of the dried material.

Supercritical carbon dioxide (CO2) is commonly employed in supercritical fluid drying due to its relatively low critical temperature of 31.5 °C, significantly lower than water's 374.4 °C. Additionally, the Food and Drug Administration recognizes it as a safe substance for food treatment applications. However, research in this drying area is somewhat limited, and potential issues such as residual CO2 in the product, which may alter the pH of the end product, need further investigation [213]. SCF drying is primarily utilized for drying foods and biopharmaceuticals where preserving the structures of the material pores is not critical [216]. Some notable patented applications of SFD in drying foods and biopharmaceuticals are detailed in Table 7.

**Table 7:** Applications of SCD in Foods and biopharmaceuticals

**Compounds**	**SC-Solvents**	**Reference**
Protein, peptides, nucleic acids, bacterial cells, antibodies, serums, liposomes, and viruses	Near supercritical CO_2_	[217–222]
β-Carotene, α-tocoferol and rosmarinic acid	Supercritical CO_2_	[223]
Strawberries (ascorbic acid, anthocyanins)	Supercritical CO_2_	[224]
drug substance, liposome	CO_2_ or other gases /co-solvent (ethanol)	[225, 226]
Theophylline	ethanol/CO_2_	[227]
Phenolic compounds (gallic acid resveratrol)	Supercritical CO_2_	[228]
Salmon calcitonin	Supercritical CO_2_	[229]
Insulin	Supercritical CO_2_	[230]
Fenofibrate particles	Supercritical CO_2_ + ethanol	[231]
Green tea extract	Supercritical CO_2_	[232, 233]

While supercritical fluid drying presents a new approach for drying functional foods and biopharmaceuticals, several challenges persist. These include the need for high pressure, potential residual solvents in the product, and substantial investment for setup and operation.

In conclusion, "Drying System for Heat-Sensitive Biomaterials" section provided a comprehensive review of the several drying methods used in drying food and biopharmaceuticals. It is evident that the preservation and retention of the nutritional value and bioactive properties of functional foods and biopharmaceuticals during drying is an area that needs further research. Our thorough research of the published work also showcased that each reviewed method offers unique advantages and presents certain limitations, influencing its suitability for different applications. Despite the challenges associated with each method, ongoing research and development efforts are continually seeking to optimize these techniques and address their limitations. The following section will build on this foundation to explore hybrid drying methods that combine the strengths of multiple techniques, pointing toward the future of drying technology.

## Hybrid Drying Methods: Innovation and Opportunities

The effectiveness and appropriateness of the aforementioned drying methods depend on the of biomaterial or bioactive compound type, the initial state of the material to be dried, and the desired final product form and functionality. Table 8 provides a comprehensive summary of the various drying methods, highlighting their strengths and limitations and the biomaterials for which each method holds the greatest application potential. As reported in Table 8, many of these drying techniques have limitations that could be minimized by combining the different techniques to improve the overall drying process, preserving the product integrity, efficacy, and quality of biopharmaceuticals and nutraceuticals while enhancing efficiency and cost-effectiveness.

**Table 8:** A comprehensive summary of the various drying methods, highlighting their strengths and limitations and the suitability for biomolecules or bioproducts

**Drying Method**	**Suitable Biomolecules/Products**	**Benefits**	**Overall Limitation**	**References**
Heat Pump Drying	High-value foods, aquatic products preserve essential amino acids	Lower energy consumption, well-controlled temperature profiles	Regular maintenance, environmental concerns, high capital cost	[63, 234, 235]
Freeze Drying	Biopharmaceuticals, high-value functional foods and ingredients such as Gelatin, isolated proteins, probiotics	Preserves structure, biochemical and immunological characteristics	High energy and cost, ice formation, protein stability issues	[79, 80, 82, 84, 236]
Spray Drying	Microencapsulation of biopharmaceuticals and biomaterials, food additives, gelatins, active biomolecules	Rapid drying, good reconstitution capacity, suitable liquid suspensions, higher foam expansion	Sensitive to moisture, potential for product sticking, non-uniform particle size, high temperatures driven denaturation	[111, 113, 122, 237, 238]
Fluidized Bed Drying	Granular solids, phytochemicals, coating/tableting for pharmaceutical and probiotics	Rapid drying speeds, enhanced thermal efficiency	High power consumption, attrition, unsuitable for highly wet products	[131, 133, 134, 136, 239]
Superheated Steam Drying	Fruits, vegetables, herbs, aquatic products	Non-polluting, preserves quality and aroma, faster drying rates	Complex setup, risk of overcooking, expensive equipment,	[143, 148, 154, 187]
Infrared Drying	Antioxidant-rich foods, herbs, nuts and seeds, green tea, fruit peels	Reduced drying time, high heat transfer rates,	Uneven heating, high initial setup cost, limited to certain products due to browning reactions and changes to functional properties	[164, 168, 173, 183, 240]
Microwave Drying	Heat-sensitive foods, biopharmaceutical powders.	Volumetric heating, enhanced heat and mass diffusion, improved energy efficiency. Suitable for hybrid drying	Non-uniform heating, high equipment cost, shielding needed	[188, 241, 242]
Osmotic Drying	Fruits, vegetables for bioactive compounds	Minimizes loss of functional components, preserves flavor, suitable as a pre-treatment	Texture changes, nutrient leaching, time-consuming	[243–246]
Vacuum Drying	Herbs, heat-sensitive foods, fruits, formulation of proteins	High-quality dried products, retains nutritional value, Suitable for hybrid drying for bioactive compounds	Expensive equipment, slower drying times, oxidation issues	[208, 247, 248]
Supercritical Fluid Drying	Proteins, peptides, sensitive biomolecules	Effective for heat-sensitive materials, minimal nutrient loss, suitable for bioactive compound extractions and hybrid drying	High pressure, residual solvents, complex setup, scaling challenges	[215, 219, 232, 233]

The ongoing quest to retain the bioactive properties of foods and biopharmaceuticals during drying has sparked numerous innovations, including developing hybrid drying methods. By integrating two or more existing techniques, these hybrid methods are designed to leverage the strengths of each approach, thereby compensating for their limitations.

The industry and researchers increasingly recognize emerging hybrid techniques such as microwave-assisted vacuum drying, microwave sprouted bed drying, superheated steam fluidized bed drying, vacuum double-drum drying, spray freeze drying, and infrared-assisted drying in functional foods and biopharmaceuticals due to their superior efficiency and performance [8, 249, 250]. The advent of particle engineering, encapsulation, and the development of novel functional food ingredients in biopharmaceuticals have underscored the need for comprehensive research on tailored drying strategies and hybrid methods. Table 9 presents examples of hybrid drying methods and their applications in various functional foods.

**Table 9:** Hybrid drying methods for various functional foods

**Functional Food Product**	**Drying method and condition**	**Reference**
Viable Probiotics	Fluidized bed drying with encapsulation	[239]
Egg White Powder	Foam mat freeze-drying	[251]
Lactobacillus plantarum in aloe vera and agave fructans, whey protein	Spray Freeze-Drying	[238, 252, 253]
Apple pomace powder, blueberries	Microwave-assisted vacuum drying	[242, 254]
Biologics and Vaccines	Microwave Vacuum Drying	[209]
Passionflower (*Passiflora alata*)	Spray and spouted bed	[252]
Mexican plum fruit extract	Spray Drying and Spout-Fluid Bed Drying Microencapsulation	[255]
Wolfberry (*Lycium barbarum L.*)	Far-infrared radiation heating assisted pulsed vacuum drying (temperature of 65 °C, vacuum pressure for 15 min, and normal pressure for 2 min)	[256]
Pre-osmodehydrated watermelon	CO_2_ convective drying with Far-Infrared radiation heating assisted pulsed vacuum drying	[257]
Potato slices (Phenolic and Flavonoids)	Ultrasound-assisted far-infrared radiation drying (ultrasonic resonant frequency of 28 ± 0.5 kHz and temperature of 50 °C)	[258]
Pear slices (Phenolic and Flavonoids)	Contact ultrasound-assisted far-infrared radiation drying (ultrasonic resonant frequency of 28 ± 0.5 kHz and temperature of 30 °C)	[259]
Garlic Slices (allicin content)	Ultrasonic-assisted vacuum drying (ultrasonic resonant frequency of 40 kHz and temperature of 60 °C	[260]
Acai puree (anthocyanin, phenolic compounds, antioxidants)	Infrared-assisted freeze-drying	[261]
Polyphenol-enriched maple sugar	Vacuum double-drum drying (80 °C and 87.99 kPa)	[262]
Mulberry leaves extract	Supercritical fluid extraction and spray drying	[263]
Chrysanthemum cake (Phenols)	Infrared and Hot Air Drying	[264]

## Advancements and Future Directions in Drying Technologies

### Novel Drying Techniques

The novel, fourth-generation dryers primarily focus on product quality, drying efficiency, time and temperature changes. This category's major types of dryers are high-vacuum, microwave, radio-frequency, and refractive window drying [265]. Among them, microwave and radio-frequency drying have gained comparatively faster commercial applications and attention from food processors and researchers over the others. Even though the technologies using electromagnetic heating, such as radio-frequency and microwave drying, have been researched for decades, the commercial application is still lagging behind the other types. The commercial-level scale-up of radio-frequency drying is limited by the large number of parameters that control the drying efficiency, such as dielectric, physical, and thermal characteristics of the biomaterial to be dried, voltage of electrode, electrode distance, etc. All these factors result in non-uniform heating and uneven distribution of temperature [266, 267]. Hence, there is ongoing research on novel drying technologies such as halogen drying [268] and refractive window drying [269–271]. Refractive window drying has recently been researched for its specific indirect heating of the material and its potential application for low-temperature and short-time processes to dry delicate, heat-sensitive products [272]. This novel drying technique is based on all three modes of heat transfer through conduction, convection, and radiation. It is ideally suitable for liquid materials where high moisture material is spread over a thin infra-red film; the refractive indices of the water and the material become similar, reducing reflection at the interface and enhancing the transmissivity of radiant energy to the product. The method is reported to maintain product temperature between 60–70 °C due to evaporative cooling and convective heat transfer to the ambient air above the material [271, 272]. Despite the greater research and development in novel drying techniques, the commercial application of these techniques in the biopharmaceutical and nutraceutical food industries is limited by various factors such as cost, scalability, infrastructure requirement, technical expertise, etc.

### Integration of Automation and Control Technologies

The landscape of drying technologies for functional foods and biopharmaceuticals is transforming substantially by integrating intelligent automation and control technologies. As advancements in artificial intelligence (AI), machine learning, the Internet of Things (IoT), and cyber-physical systems continue, new opportunities for improving precision, efficiency, and sustainability in drying technologies are revealed.

Central to this transformation is the role of process automation, which involves using advanced software and hardware to manage and monitor drying processes. Control systems equipped with programmable logic controllers (PLCs) and supervisory control and data acquisition (SCADA) systems offer precise control over process variables such as temperature, humidity, and air velocity. These systems ensure consistent product quality while minimizing energy consumption and waste by maintaining optimal drying conditions. Furthermore, automation remarkably reduces the need for manual supervision, thereby cutting labour costs and human error [273, 274].

Implementing AI and machine learning has demonstrated its efficacy across various sectors, including biopharmaceuticals [275–277], drying technologies [278–280], and agri-food quality monitoring [281–283]. Alongside automation, incorporating AI and machine learning within drying technologies has unveiled new avenues for enhancing process efficiency. Machine learning algorithms can analyze historical and real-time data, enabling the prediction of optimal drying conditions and swift responses to changes in process variables. Likewise, predictive models serve to optimize drying schedules, reduce energy consumption, and improve product quality. Moreover, advanced control systems developed with the help of AI and profound learning neural networks can learn, adapt, and make autonomous decisions based on complex data inputs, thus managing the nonlinear and dynamic nature of drying processes [278, 280, 284–286].

The complexities and scale of modern drying processes necessitate managing and analyzing large volumes of data, a demand met by cloud computing and big data analytics. Cloud computing and big data analytics provide scalable computational resources and tools for extracting valuable insights from complex data sets. Such capabilities support advanced AI and machine learning applications, predictive modelling, and real-time process optimization [287, 288].

Smart sensors and IoT technologies complement these developments, facilitating real-time monitoring and control of drying processes. Smart sensors gather granular data on various process parameters and environmental conditions, while IoT ensures interconnectivity among these sensors, forming a comprehensive and synchronized data network [285, 287, 289, 290]. This real-time data fuels AI and machine learning systems, empowering predictive analytics, real-time adjustments, and proactive maintenance. Moreover, IoT integration can extend beyond individual drying systems to include entire production lines or even multiple manufacturing sites, fostering systemic efficiency and coherence.

Advancing this integration further, cyber-physical systems (CPS) represent the next fusion level between hardware and software in drying technologies. These systems tightly couple the computational (cyber) elements with the physical components of a drying process. Creating a digital twin – a real-time virtual replica of the physical process – is possible with CPS. Such digital twins can simulate and evaluate different process conditions and control strategies, yielding substantial improvements in system design and operation [291–294].

Ultimately, these integrated advancements in automation, AI, machine learning, IoT, and cyber-physical systems drive the evolution of drying technologies for functional foods and biopharmaceuticals. The continued exploration of these cutting-edge technological advancements will further shape and enhance the efficiency and sustainability of drying processes.

### Trends and Research Directions

**Fig. 3:**
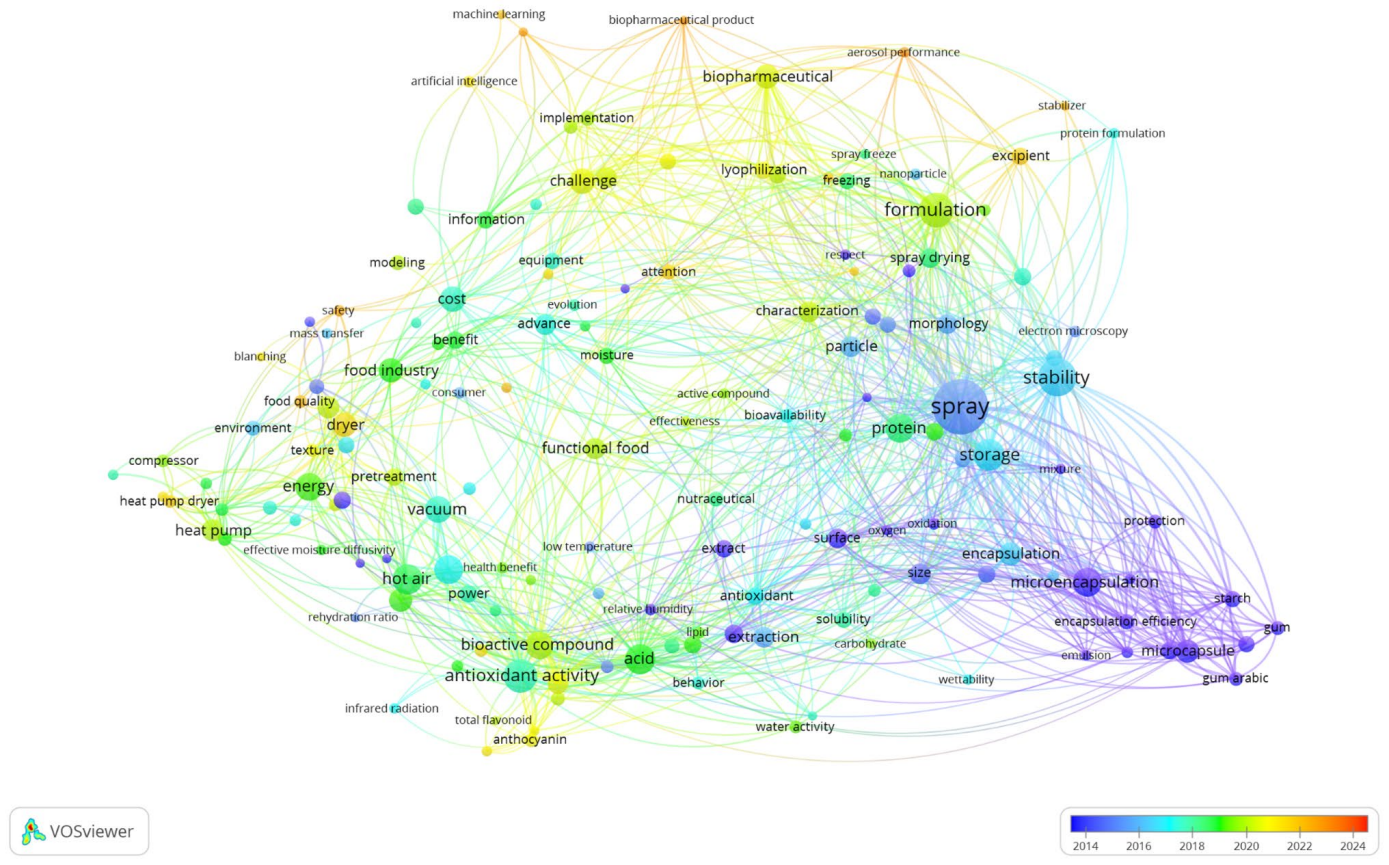
Network visualization map on advancement and innovations landscape in drying of biopharmaceuticals, nutraceuticals, and functional foods

Drying technologies for functional foods and biopharmaceuticals have made remarkable strides, yet the future has potential for further innovation and refinement. A fundamental challenge lies in developing drying techniques that balance energy efficiency, cost-effectiveness, scalability, and preservation of nutritional and bioactive properties. To discern patterns and trends of advancement and innovations in the drying of biopharmaceuticals, nutraceuticals, and functional foods, a network visualization map was generated using VOSviewer as shown in Fig. 3. The figure visualizes the trends in drying of biopharmaceuticals, nutraceuticals, and functional foods over the years classified with keywords/terms with a technique of full counting generating 5 clusters of keywords. Based on the network visualization map, the advancement and innovations landscape in the drying of biopharmaceuticals, nutraceuticals, and functional foods showed that the earlier years of studies and focus were more skewed to the application of spray drying and micro-encapsulated spray-drying for biopharmaceuticals, nutraceuticals, and functional foods. This trend in technology and research has turned more towards hybrid drying systems and the application of machine learning, AI and IoT technology for improved hybrid drying systems for better drying efficiency and techno-functional properties of the final products in recent years.

In the meantime, as discussed in "Advancements and Future Directions in Drying Technoligies" section, the digital age allows further integration of advanced technologies into these drying methods. Artificial intelligence, machine learning, and smart sensor technologies can profoundly transform drying processes. These technologies enable superior control, permit real-time modifications, and facilitate comprehensive optimization of drying processes, effectively ushering in a new era of intelligent and responsive drying techniques. These technologies use advanced sensors, data analytics, automation, and connection to improve drying efficiency, reduce energy consumption, and maintain product quality and safety. Preliminary studies on the IoT-based control system for smart drying technologies demonstrated the potential to preserve food's functional qualities and nutraceutical values, such as rehydration capacity, crude fibre, protein, and vitamin C levels, etc., compared to conventional drying method counterparts [295].

**Fig. 4:**
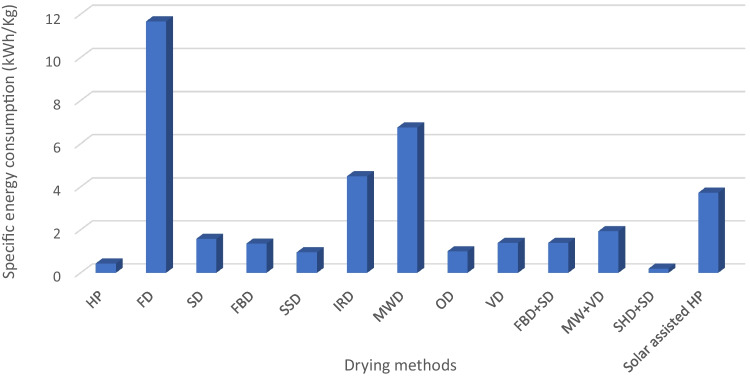
Comparison of specific energy consumption (SEC) for different drying techniques [161, 174, 245, 296–299]

In parallel with technological innovations, the shift towards sustainable production systems necessitates a thorough understanding of the environmental impact and sustainability of drying techniques. This includes an evaluation of energy consumption, water usage, waste production, and how these techniques align with evolving regulatory requirements worldwide. The specific energy consumption (SEC) of drying technologies refers to the energy required to remove a unit of moisture from a product. Even though SEC is considered a good indicator of the energy performance of drying methods, it is often not proportional to the techno-functionality of this drying technology application in biopharmaceuticals and nutraceuticals. Figure 4 compares available data on SEC of different drying methods [296]. For instance, freeze-drying (lyophilization) is often preferred for biopharmaceuticals and certain nutraceuticals despite its relatively high energy consumption compared to other drying methods (Fig. 4). It has been reported to have a lower specific energy consumption (SEC) than freeze-drying, although it reduces total phenolic compounds [174]. Consequently, a hybrid infrared-freeze drying method has been reported effective for bioactive compounds to combine the benefits of both techniques, ensuring quality and energy efficiency [172, 174, 261]. Therefore, the choice of drying technology involves a trade-off between energy efficiency (as measured by SEC) and other factors such as product quality, safety, and regulatory compliance.

From an economic perspective, detailed analyses are needed to evaluate the financial aspects of various methods as a function of techno-functionality. These would consider elements such as capital and operating costs and return on investment, providing insights into the economic feasibility of each method. This information would guide industries in making informed decisions on adopting these drying techniques.

Lastly, it is crucial to continue exploring the challenges and limitations inherent in different drying techniques, the potential integration of drying techniques for enhanced quality and sustainability, and the dedication of research efforts to finding potential pathways for digital transformation in the automation of these drying systems. This exploration will shape the evolution of drying technology, ensuring its practical viability and suitability for both functional foods and biopharmaceuticals.

Looking ahead, the emphasis should be on developing drying techniques that are not only efficient and sustainable but also economically viable. These methods must retain the quality of end products, ensuring that the therapeutic and nutritional benefits remain intact. Achieving harmony among these aspects will be pivotal in shaping the future landscape of drying techniques in the food, nutraceutical, and biopharmaceutical industries.

## Conclusion

The process of drying functional foods and biopharmaceuticals poses unique challenges to industries due to the heat-sensitive nature of these products. Consequently, selecting the appropriate drying strategy and methods requires careful consideration, as different products require varying initial conditions to maintain their bioactive and functional components. Ongoing research focuses on enhancing existing systems and designing innovative hybrid solutions to improve drying outcomes. Functional foods and biopharmaceuticals are commonly dried under controlled conditions - either at lower temperatures or higher temperatures for brief periods - to safeguard the intrinsic functional properties. Critical to this process is a deep understanding of particle engineering for optimal rheology and microstructure and devising product-specific drying strategies. This understanding is fundamental given that several chemical instabilities, such as oxidation, aggregation, chemical bonding, and glycation, are commonplace in biomolecules. As such, optimizing various drying methods, including freeze-drying, vacuum drying, and superheated steam drying techniques, is essential for each category of these products, necessitating a comprehensive study of their nutritional and functional properties. The ongoing evolution of drying techniques is pivotal for the future of functional foods and biopharmaceuticals, seeking to balance quality retention, efficiency, and industrial feasibility in an ever-changing landscape. This comprehensive account of the advantages and limitations of each commonly used drying method provides researchers with a critical first building block to devise future innovative modifications to push the state-of-the-art into its future for drying products rich in bioactive volatiles.

## Data Availability

Not applicable
